# Effect of recombinant bovine somatotropin (rbST) treatment on follicular population and development in non-lactating dairy cows

**DOI:** 10.21451/1984-3143-AR2018-0118

**Published:** 2019-11-18

**Authors:** Joao Alveiro Alvarado Rincón, Bruna Mion, Diego Andres Velasco Acosta, Bernardo Garziera Gasperin, Monique Tomazele Rovani, Lígia Margareth Cantarelli Pegoraro, Marcio Nunes Corrêa, Augusto Schneider

**Affiliations:** 1 Faculdade de Veterinária, Universidade Federal de Pelotas, Pelotas, RS, Brasil; 2 Corporación Colombiana de Investigación Agropecuaria, Mosquera, Cundinamarca, Colombia; 3 Instituto Federal Farroupilha, Frederico Westphalen, RS, Brasil; 4 Embrapa Clima Temperado, Empresa Brasileira de Pesquisa Agropecuária, Pelotas, RS, Brasil; 5 Faculdade de Nutrição, Universidade Federal de Pelotas, Pelotas, RS, Brasil

**Keywords:** follicular population, gene expression, steroidogenesis

## Abstract

The aim of this study was to evaluate the long-term effects of recombinant bovine somatotropin (rbST) on follicle population and ovulatory follicle development in non-lactating dairy cows. Twenty-one Jersey cows were allocated in rbST (n=11) or control (n=10) groups. On day -60, cows in rbST group received 500 mg of somatotropin (s.c. Lactotropin®, Elanco). On day 0, control and rbST cows received an intravaginal progesterone-releasing device (1.9 g, CIDR^®^, Zoetis) and GnRH (100 mg, IM, Factrel^®^, Zoetis). On day 8, cows received PGF2α (25 mg, IM, Lutalyse®, Zoetis) and the CIDR^®^ was removed. Twelve hours after device removal (D8), serum, follicular fluid and granulosa cells samples were collected. Serum and follicular concentration of estradiol (E2) and progesterone (P4) were analyzed. Total RNA was extracted from granulosa cells to measure gene expression of *LHCGR*, *STAR*, *HSD-3B1*, *CYP11A1*, *CYP19A1*, *CYP17A1*, *IGFR* and *PAPPA* by real-time PCR. Ultrasonography was performed on days -60, -53, -46, -14, -7, 0 and 8 for antral follicle count. Results were analyzed by repeated measures ANOVA and t-test. There was no effect of rbST treatment on the number of follicles during the 60 days period, as well as no effect on serum and follicular fluid E2 and follicular fluid P4 at the moment of follicle aspiration. There was a reduction in *PAPPA* (*P* = 0.006), *CYP11A1* (*P* = 0.04) and *CYP19A1* (*P* = 0.002) mRNA levels in granulosa cells of the pre-ovulatory follicle of rbST treated cows. In conclusion, a single dose of rbST did not have long-term effects on antral follicle population, serum and follicular E2/P4 concentrations in non-lactating dairy cows. Despite that, rbST injection decreased granulosa cell expression of genes related to steroidogenesis in the pre-ovulatory follicle.

## Introduction

Over the past years, aiming to improve the reproductive efficiency of dairy cattle, several strategies involving mainly nutrition management (Roche, 2006) and protocols for ovulation induction have been studied (Thatcher et al., 2002; Lucy, 2007). Both the duration and intensity of the postpartum negative energy balance (NEB) are negatively associated with reproductive performance (Butler and Smith, 1989). The growth and estradiol (E2) production by the first postpartum dominant follicle are key factors for a successful ovulation (Butler et al., 2006; Kawashima et al., 2006), and its impairment can be attributed to reduced luteinizing hormone pulses (Grainger et al., 1982) as well as decreased circulating insulin and insulin-like growth factor I (IGF-I) concentrations (Butler et al., 2006; Kawashima et al., 2007). In the early postpartum period, increased growth hormone (GH) and decreased insulin concentration facilitate adipose tissue mobilization and support milk production (Lucy et al., 2001). The metabolic status associated to NEB decreases the expression of liver GH receptor and, consequently, decreases serum IGF-I-concentration (Butler et al., 2003). In this context, cows ovulating the first postpartum dominant follicle have higher serum IGF-I concentration than cows with non-ovulatory follicles (Taylor et al., 2004; Butler et al., 2006). IGF-I acts as modulator of gonadotropin action in the ovary, stimulating proliferation and differentiation of granulosa and theca cells (Armstrong and Webb, 1997). Therefore, strategies have been studied to minimize the effects of NEB and increased serum IGF-I to anticipate the moment of the first postpartum ovulation and improve dairy cow reproductive performance.

Treatment with exogenous recombinant bovine somatotropin (rbST) is usually performed during the postpartum period, aiming to enhance efficiency of milk synthesis (Bauman, 1999) and improve persistence of lactation (Bauman, 1992). Peripartum rbST treatment promotes an increase in serum IGF-I concentration (Carriquiry et al., 2009) and glucose, and a decreased serum non-esterified fatty acids and beta-hydroxybutyrate concentration (Putnam et al., 1999). Despite positive effects on lactation, some studies suggest that rbST negatively affects reproductive performance in dairy cows in the long term (Cole et al., 1992; Kirby et al., 1997b; Santos et al., 2004). Interestingly, the prepartum treatment with rbST was associated with a shorter interval between calving and first ovulation in dairy cows (Schneider et al., 2012). Prepartum rbST treatment increased production of E2 and expression of key steroidogenic enzymes by the first postpartum dominant follicle (Acosta et al., 2017). These results suggest a long-term effect of rbST on ovarian function depending in what stage of lactation is used that is still not totally understood.

Previous studies have demonstrated that the GH/IGF-I axis is essential for the recruitment of primordial follicles in mammals (Saccon et al., 2017) and for the growth of larger follicles in cows (Shimizu et al., 2008). In this context, treatment of cows and heifers with rbST increased the number of small antral follicles (Buratini et al., 2000), follicles class 1 (3-5 mm) (Gong et al., 1991; Kirby et al., 1997a); class 2 (6-9mm) (Kirby et al., 1997a; Kassa et al., 2002) and class 3 (>10mm) (De la Sota et al., 1993; Kirby et al., 1997a). These studies suggest a role for the GH/IGF-I axis in increasing antral follicle population in cows. Follicular growth from early pre-antral stages up to ovulatory size requires approximately 60 to 80 days in cows (Lussier et al., 1987; Britt, 1991). Therefore, rbST treatment can have long-term effects on ovarian physiology, through increased recruitment of pre-antral follicles. Furthermore, evidence suggests that a higher antral follicular count in dairy cows is associated with healthier antral follicles (Ireland et al., 2008) and increased fertility (Martinez et al., 2016).

Based on these evidences the GH/IGF-I axis may play and indirect role in ovulatory follicle development through increased antral follicle population. However, previous reports indicate a negative effect of GH/rbST on reproduction. Most studies with rbST were performed with lactating dairy cows for obvious practical reasons, however lactation can be a confounding factor in these studies, as rbST treatment increases milk production (Bauman, 1999). rbST intensifies NEB and has negative effects on the reproductive performance, suggesting that testing its effects on non-lactating cows could help better understand its physiology. Based on this, the aim of this study was to evaluate the long-term effect of rbST administration on follicle population and development of the ovulatory follicle in non-lactating dairy cows. Our hypothesis is that rbST can increase preantral follicle growth and recruitment and consequently have long-term beneficial effects on ovulatory follicle development.

## Methods

All procedures performed in this experiment were approved by the Committee for Ethics in Animal Experimentation from the Federal University of Pelotas, protocol 4006-2015 (Pelotas, RS, Brazil).

### Animals and treatments

The study was performed with non-lactating Jersey dairy cows (n=21), kept in pasture. The cows had body condition score (BCS) between 2.5 and 3.5 (on a scale of 1 to 5) and were randomly (by BCS) assigned to two treatments: control (n=10) and somatotropin (rbST, n=11). The rbST group received one injection of somatotropin (500 mg/cow, s.c., Lactotropin®, Elanco, SP, Brazil) 60 days before the beginning of synchronization protocol (Day -60). On day 0, all cows received an intravaginal progesterone (P4)-releasing device (1.9 g, CIDR®, Zoetis, NJ, USA) and a GnRH injection (100 mg, IM, Factrel®, Zoetis). On Day 8, at the moment of P4 device withdrawal, all cows received an injection of PGF2α (25 mg, i.m., Lutalyse®, Zoetis). Transrectal ultrasonography (7.5 MHz linear array probe, Aquila pro, Esaote, São Paulo, SP, Brazil) was performed on days -60, -53, -46, -14, -7, 0 and 8 for antral follicle count. All follicles detected by ultrasonography were counted and classified according to its diameter in three categories: Class 1 (<6 mm), Class 2 (6-9 mm) and Class 3 (>9 mm). On day 8 of the protocol, the diameter of the largest follicle was measured through ultrasonography.

### Serum, follicular fluid and granulosa cells collection

Twelve hours after removal of the intravaginal P4 device cows received an epidural anesthesia (3 ml of 2% lidocaine) and the largest follicle aspirated by transvaginal ultrasound-guided aspiration (5.0 MHz convex transducer, Aquila Pro, Esaote). The follicular fluid and granulosa cells were recovered according to a previously described procedure (Schneider et al., 2013). Follicular fluid was centrifuged at 1500 × G for 10 minutes and the clear follicular fluid was stored at -80°C for subsequent analysis. The pellet of granulosa cells was re-suspended in Trizol (Life Technologies, Carlsbad, CA, USA), homogenized and stored in liquid nitrogen.

Blood samples were collected by coccygeal venipuncture at the moment of follicle aspiration. Serum was obtained by centrifugation at 1000 × G for 15 minutes and stored at -80°C for analysis of E2 and P4 concentrations.

### Hormonal analysis of serum and follicular fluid

Serum and follicular fluid concentration of E2 and P4 were analyzed in a commercial laboratory by chemiluminescence methods (Laboratório Pasin, Santa Maria, RS, Brazil). Follicular fluid samples were diluted 1:500 before E2 analysis. Two samples (one from control group and one from rbST group) were excluded from further analysis since intrafollicular E2:P4 ratio was lower than 1, indicating that an atretic follicle was aspirated (Ireland and Roche, 1982; Cheong et al., 2016).

### Analysis of gene expression

Total RNA was extracted from granulosa cell using the guanidine isothiocyanate protocol (Trizol, Life Technologies) following the manufacturer recommendations. The RNA was quantified in a spectrophotometer (Nanodrop Lite, Thermo Fischer Scientific Inc., USA) and adjusted to a concentration of 200 ng/μL. Samples with a 260/280 nm absorbance ratio below 1.8 were excluded. Reverse transcription was performed with 1 μg of total RNA in a volume of 20 μL using the iScript Synthesis Kit (BioRad, Hercules, CA, USA) according to manufacturer instructions. Real time quantitative PCR was performed in duplicate in a final volume of 20 μL, using SYBR Green Mastermix (Applied Biosystems, Foster City, CA, USA), 10 μM of each primer and 20 ng of cDNA. For each assay, 45 cycles (95°C for 10 seconds and 60°C for 30 seconds) were run in the Real-Time ECO PCR system (ILLumina, San Diego, CA, USA) and a dissociation curve was included at the end of reaction to detect the specificity by the amplification of a single PCR product.

The H2A Histone Family Member Z (*H2AFZ*) gene was used as internal control and the target genes evaluated were: Luteinizing Hormone/Choriogonadotropin Receptor (*LHCGR*), Steroidogenic acute regulatory protein (*STAR*), Cytochrome P450 Family 11 Subfamily A Member 1 (*CYP11A1*), Cytochrome P450 Family 19 Subfamily A Member 1 (*CYP19A1*), Cytochrome P450 Family 17 Subfamily A Member 1 (*CYP17A1*), 3 beta-hydroxysteroid dehydrogenase (*HSD-3B1*), pappalysin 1 (*PAPPA*) and IGF-I receptor (*IGFR*). The respective primer sequences are described in Table 1.

**Table 1:** Genes and primer sequences analyzed in this study.

**Gene**	**Sequence 5ʹ → 3ʹ**	**Reference**
*H2AFZ*	F: GAGGAGCTGAACAAGCTGTTG	Portela et al. (2010)
	R: TTGTGGTGGCTCTCAGTCTTC	
*LHCGR*	F: TGACTATGGTTTCTGCTTACCCAA	Spicer et al. (2008)
	R: CCATAATGTCTTCACAGGGATTGA	
*STAR*	F: TCGCGGCTCTCTCCTAGGT	Spicer et al. (2008)
	R: CTGCCGGCTCTCCTTCTTC	
*CYP11A1*	F: CTTCATCCCACTGCTGAATCC	Tajima et al. (2005)
	R: GGTGATGGACTCAAAGGCAAA	
*CYP19A1*	F: TGCCAAGAATGTTCCTTACAGGTA	Spicer et al. (2008)
	R: CACCATGGCGATGTACTTTCC	
*CYP17A1*	F: GAATGCCTTTGCCCTGTTCA	Buratini et al. (2005)
	R: CGCGTTTGAACACAACCCTT	
*HSD-3B1*	F: CCAAGCAGAAAACCAAGGAG	Nishimura et al. (2006)
	R: ATGTCCACGTTCCCATCATT	
*PAPPA*	F: TGGAGAACGCTTCGCTCAACTG	Rodríguez et al. (2015)
	R: ACGCTGGGTCCTGTCTGGCTTT	
*IGFR*	F: CACGCCTTGGTCTCCTTGTCCT	Rodríguez et al. (2015)
	R: CGTCACTTCCTCCATGCGGTAAAT	

The coefficient of variation was less than 2% for all primer pairs used. Relative expression was calculated using the 2^A-B^/2^C-D^ equation (where A is the cycle threshold [Ct] number for the gene of interest in the first control sample, B is the Ct number for the gene of interest in the analyzed sample, C is the Ct number for *H2AFZ* in the first control sample, and D is the Ct number for *H2AFZ* in the analyzed sample). The first control sample is a random reference sample from the control group and its value was 1.00 as expressed by this equation, and all other samples were calculated relative to this value. After that, all other results from the rbST group were divided by the mean relative expression of all the samples in the control group, which averaged 1.00 by this equation, to obtain the relative expression of genes of interest for the rbST compared to the control group.

The *CYP17A1* gene is exclusively expressed in the theca interna cells and was used as an indicator of theca cell contamination in granulosa samples. Therefore, samples with Ct values lower than 30 for *CYP17A1* were considered as contaminated and excluded from further gene expression analysis (Buratini et al., 2005). No sample was excluded according to this criteria.

### Statistical analysis

Statistical analysis was performed using GraphPad Prism 5 software (GraphPad Software Inc., La Jolla, CA, USA). Continuous variables (i.e. gene expression, total follicular count, follicular diameter, serum and follicular concentration of E2, P4 and E2:P4 ratio) were analyzed using the Student’s T test. Follicular count of each follicular category of follicles (Class 1, 2 and 3) were analyzed using two-way ANOVA. *P* values lower than 0.05 were considered as significant. Results are presented as mean ± standard error of the mean.

## Results

**Figure 1:**
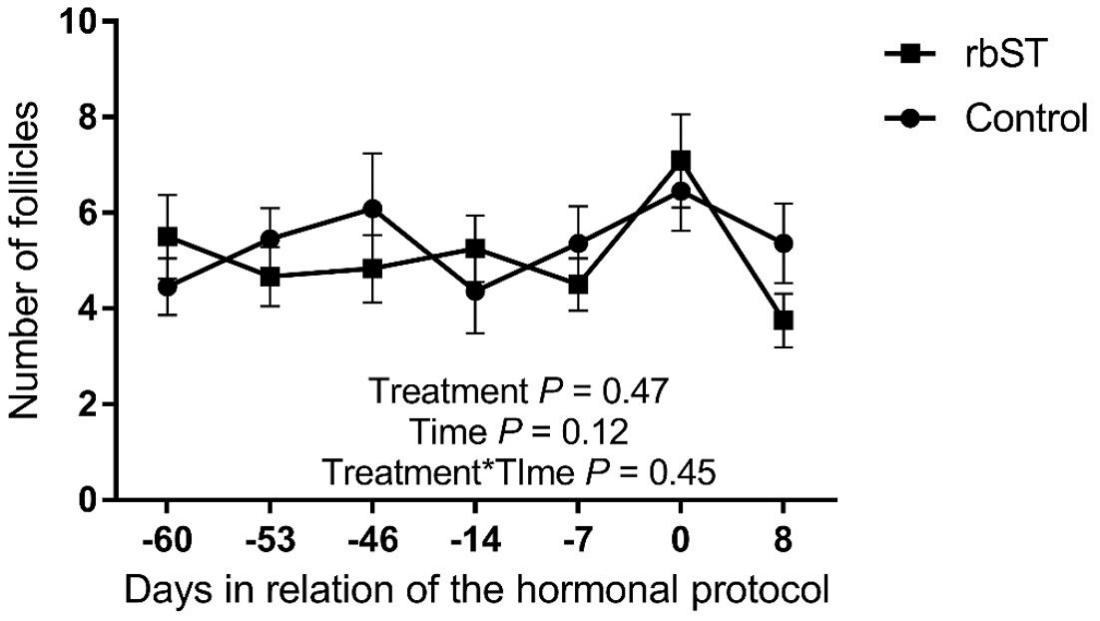
Total follicular count at different moments for recombinant bovine somatotropin (rbST) and control groups. rbST injection was performed 60 days before the beginning of the protocol. Values are presented as mean ± standard error and the differences (*P* < 0.05) are represented by different lowercase letters.

There was no effect of treatment or treatment by time interaction on the number of total follicles (*P* > 0.05, Figure 1), control group had 5.36 ± 0.31 follicles/cow and the bST group 5.08 ± 0.28 follicles/cow on average along the 60 days of experiment. Follicles were classified by size and there was no effect of rbST treatment on Class 1 (<6 mm; control: 3.54 ± 0.32 and bST: 3.05 ± 0.27 follicles; *P* = 0.29), Class 2 (6-9 mm; control: 0.70 ± 0.04 and bST: 1.07 ± 0.15 follicles; *P* = 0.56) and Class 3 follicles (>9 mm; control: 1.09 ± 0.08 and bST: 1.02 ± 0.12 follicles; *P* = 0.92, Table 2). Moreover, the diameter of the aspirated follicle after the synchronization protocol at the end of the study was not different between control (15.02 ± 1.16 mm) and rbST groups (16.47 ± 1.63 mm, *P* = 0.48).

**Table 2:** Mean number of follicles from Class 1 (<6 mm), 2 (6-9 mm) and 3 (>9mm) in cows receiving recombinant bovine somatotropin (rbST) and control. Values are presented as mean ± standard error and the differences (*P* < 0.05) are represented by different lowercase letters.

**Group**	**Follicular category**		
	Class 1	Class 2	Class 3
Control	3.54 ± 0.32	0.70 ± 0.04	1.09 ± 0.08
rbST	3.05 ± 0.27	1.07 ± 0.15	1.02 ± 0.12

Mean ± SEM.

**Figure 2:**
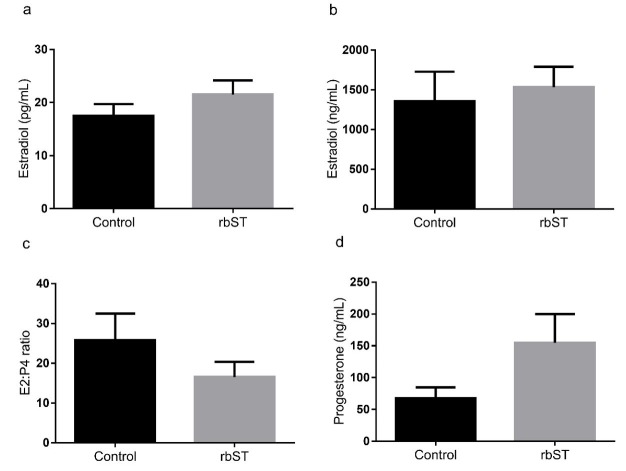
a) Serum estradiol (E2) concentration, b) Follicular E2 concentration, c) Follicular E2:P4 ratio and d) Follicular progesterone (P4) concentration for cows treated with recombinant bovine somatotropin (rbST) or control twelve hours after removal of the intravaginal P4. rbST injection was performed 60 days before the beginning of the protocol. Values are presented as mean ± standard error and the differences (*P* < 0.05) are represented by different lowercase letters.

At the moment of P4 device removal in the synchronization protocol, serum P4 concentration was less than 1 ng/mL in both groups (Control: 0.50 ± 0.09 and bST: 0.47 ± 0.79 ng/mL), indicating successful luteolysis and no levels of P4 that could interfere with follicle growth (Ginther and Beg, 2012). At the moment of follicle aspiration there was no difference between control and rbST group for E2 concentration in serum (17.5 ± 2.2 and 21.5 ± 2.6 pg/mL, respectively, *P* = 0.27). Additionally, the concentration of E2 (1356 ± 371.3 and 1532 ± 256.5 ng/mL, respectively, *P* = 0.71) and P4 (67.8 ± 16.9 and 154.6 ± 45.3 ng/mL, respectively, *P* = 0.08) in the follicular fluid of the largest follicle after the synchronization protocol was not different between groups. There was also no difference in the follicular fluid E2:P4 ratio of the largest follicle aspirated (25.9 ± 6.6 and 16.6 ± 3.8, respectively, *P* = 0.26, Figure 2).

**Figure 3:**
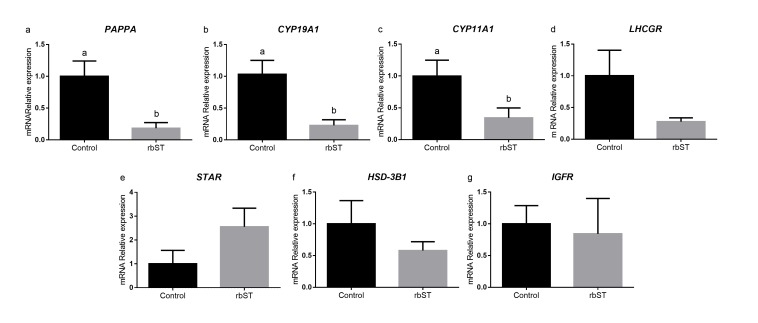
Pre-ovulatory follicle granulosa cell gene expression for cows treated with recombinant bovine somatotropin (rbST) or control twelve hours after removal of the intravaginal P4. rbST injection was performed 60 days before the beginning of the protocol. Values are presented as mean ± standard error and the differences (*P* < 0.05) are represented by different lowercase letters.

Regarding granulosa cells gene expression in the pre-ovulatory follicle aspirated, cows treated with rbST had lower expression of the IGFBP cleaving *PAPPA* enzyme (*P* = 0.006), and steroidogenic enzymes, *CYP11A1* (*P* = 0.04) and *CYP19A1* (*P* = 0.002). There was no difference between groups for *LHCGR*, *STAR*, *HSD-3B1* and *IGFR* gene expression (Figure 3).

## Discussion

The present study investigated long-term effects of rbST treatment on follicular-function in cows. Our main finding suggests that rbST treatment did not affect follicular population or development in the long term. However, there was a reduction in gene expression of steroidogenic enzymes in granulosa cells from pre-ovulatory follicles after the end of the treatment. These findings suggest a long-term effect of rbST on follicle maturation. Although an effect on important genes involved with steroidogenesis was detected, no direct impact of the treatment on estrogen production was observed.

Several studies shown that rbST treatment increases small follicles population in cows and heifers (Gong et al., 1991; De la Sota et al., 1993; Kirby et al., 1997a; Jimenez-Krassel et al., 1999; Buratini et al., 2000; Kassa et al., 2002). However, in our current study, a single rbST injection was not able to affect the number of follicle or its development. Most studies mentioned before performed more than one rbST injection, only Buratini et al. (2000) also performed a single rbST injection. However, the effect of this single rbST injection on follicular population was evaluated until 11 days after treatment, while we evaluated until 60 days after the rbST injection. We understand that a longer period is needed to evaluate the effect of rbST injection on follicular recruitment, as the follicular development from pre-antral to antral stages requires approximately 40 days in cows (Lussier et al., 1987). Therefore, the evaluation of dominant follicle growth 60 days after rbST injection was performed aiming to select an ovulatory follicle that was in the preantral stages of development at the moment of rbST injection. The long-term effect of rbST injection on follicular response was also evaluated by Kassa et al. (2002). In this study, the rbST effect was evaluated up to 58 days and a reduction in recruitment of Class 2 follicles was observed, suggesting that rbST treatment stimulates greater follicle dominance. However, in that study, fat supplementation was also performed and could interfere with results. It is still unclear how rbST treatment alone can affect follicular population in the long term. Despite that, our current study suggests no significant effect on long-term follicle population after a single rbST injection in cows.

Previous studies showed that rbST treatment in postpartum dairy cows can negatively affects estrous expression and reproductive performance (Cole et al., 1992; Kirby et al., 1997a; Santos et al., 2004). Nevertheless, prepartum rbST treatment improved ovulation and steroidogenesis of the first postpartum dominant follicle in dairy cows (Schneider et al., 2012; Acosta et al., 2017). In these studies, the direct effects of rbST on the ovaries were always influenced by the indirect effect of rbST on adaptation to lactation and negative energy balance. Therefore, we hypothesized that similar changes on steroidogenesis of pre-ovulatory follicles of rbST treated non-lactating cows in would be observed the current study. However, no difference was observed in ovulatory follicle growth pattern and concentration of E2 and P4 in serum or follicular fluid of rbST treated cows. Other studies also did not find an effect of rbST treatment in E2 concentrations (Gong et al., 1991; Kirby et al., 1997a; Jimenez-Krassel et al., 1999). Nevertheless, Rivera et al. (2010) observed a tendency of rbST treated cows to have higher concentration of E2 by the end of a FTAI protocol. De la Sota et al. (1993) also observed higher E2 concentration in the first 12 days of rbST treatment, but this difference disappeared after the end of treatment. Others reported that rbST treatment decreased the size of estrogen-active follicles during treatment (Jimenez-Krassel et al., 1999). Based on these evidences, it is still unclear how rbST can affect long term follicular development. In our study, rbST treatment did not affect small follicles population and this could explain also the lack of effect on ovulatory follicle growth and steroid production long after the end of treatment. GH has many functions in different reproductive tissues (Carlsson et al., 1992; Silva et al., 2009) we have to consider that GH effects in other organs and tissues (pituitary, uterus, CL, among others) may affected the response hypothesized for small pre-antral follicles.

The CYPs are members of the cytochrome P450 superfamily and are necessary for conversion of cholesterol into steroids precursors (Bao and Garverick, 1998) and production of estrone, estriol and E2 (Neunzig et al., 2017). We observed reduced expression of *CYP11A1* and *CYP19A1* in the granulosa cells of the pre-ovulatory follicles of rbST treated cows, suggesting that rbST had a negative impact on steroidogenesis pathways. Similarly, the reduction of *PAPPA* expression in granulosa cells from rbST treated cows can lead to decreased follicular IGF-I bioavailability. In the ovary, IGF-I bioavailability is regulated by insulin-like growth factor-bindind proteins (IGFBPs) (Yakar and Adamo, 2012) and PAPPA (Sudo et al., 2007). The intrafollicular cleavage of IGFBP4 performed by PAPPA can increase IGF-I bioavailability and stimulate granulosa cell proliferation and steroidogenesis (Mazerbourg et al., 2001). In this sense, decreased PAPPA secretion by granulosa cells of rbST treated cows could decrease steroidogenesis. This observation, added to the reduced granulosa cell *CYP11A1* and *CYP19A1* expression in rbST treated cows, suggests a reduced ability of the pre-ovulatory follicle to produce E2 (Neunzig et al., 2017). However, we did not difference in follicular E2 and P4 concentrations. This could be due the short interval between P4 device removal and follicle aspiration, which did not allow enough time to reflect these differences in the intrafollicular accumulation of E2 and P4. Overall, our findings are in alignment with previous observations that rbST can reduce estrous response in postpartum dairy cows (Kirby et al., 1997a; Santos et al., 2004).

Poor reproductive performance postpartum is associated with a more severe NEB, greater insulin resistance, fewer LH pulses and lower follicular fluid androstenedione and E2 concentration (Cheong et al., 2016). Previous studies suggested that administration of rbST could help improve reproductive performance due its positive effect in glucose metabolism (Gohary et al., 2014) and serum IGF-I concentration during the development of the first postpartum follicular wave (Schneider et al., 2012; Silva et al., 2017). It is well know in dairy cows that the early postpartum severe NEB is associated with reduced IGF-I synthesis (Fenwick et al., 2008). However, we in our study we used non-lactating cows that were not in NEB, which could attenuate the positive effects of rbST in the ovarian physiology. Our results suggest that no significant improvement in reproductive performance should be expected when associating rbST injection to FTAI protocols in non-lactating cows.

In conclusion, a single injection of rbST in non-lactating dairy cows did not have long-term effects on follicle population, serum and follicular fluid E2 and P4 concentrations in the pre-ovulatory follicle. However, rbST has decreased expression of genes related to the synthesis of the steroid hormones by the granulosa cells of the pre-ovulatory follicle.
